# Feasibility of fast, four-dimensional computed tomography-based O-ring LINAC plans for lung stereotactic body radiotherapy in patients with poor performance status

**DOI:** 10.3389/fonc.2023.1270677

**Published:** 2023-11-20

**Authors:** Eun Jeong Heo, Minseok Kim, Chun Gun Park, Kyung Hwan Chang, Kwang Hyeon Kim, Jang Bo Shim, Young Je Park, Chul Yong Kim, Nam Kwon Lee, Suk Lee

**Affiliations:** ^1^ Department of Radiation Oncology, College of Medicine, Korea University, Seoul, Republic of Korea; ^2^ Department of Medical Physics, Graduate School of Korea University, Sejong, Republic of Korea; ^3^ Department of Biostatistics and Computing, Yonsei University Graduate School, Seoul, Republic of Korea; ^4^ Department of Mathematics, Kyonggi University, Suwon-si, Gyeonggi, Republic of Korea; ^5^ Department of Radiologic Science, Far East University, Eumseong-gun, Chungcheongbuk-do, Republic of Korea; ^6^ Department of Neurosurgery, Ilsan Paik Hospital, College of Medicine, Inje University, Goyang-si, Gyeonggi-do, Republic of Korea; ^7^ Department of Radiation Oncology, Guro Hospital, Korea University Medical Center, Seoul, Republic of Korea

**Keywords:** fast treatment, O-ring LINAC, poor performance status, 4D-SBRT, lung cancer

## Abstract

**Purpose:**

We aimed to retrospectively analyzed the feasibility of fast four-dimensional computed tomography (4DCT)-based O-ring LINAC treatment for patients with an average respiratory amplitude was< 0.5 cm and who cannot endure long treatment times due to poor performance status in lung 4D-stereotactic body radiotherapy (SBRT).

**Methods:**

This study included data of 38 patients who received lung 4D-SBRT and had average respiratory amplitude< 0.5 cm in the full phase. C-arm LINAC plans were based on 4DCT data obtained at phase values ranging from 20–70% using a C-arm LINAC. O-ring LINAC plans were retrospectively established based on 4DCT data obtained at phase values of 0–90% using an O-ring LINAC. The conformity index (CI), homogeneity index (HI), and gradient measurement of the planning target volumes (PTV) were analyzed to compare dosimetric data between C-arm LINAC and O-ring LINAC plans. Organs at risk were analyzed in accordance with the Radiation Therapy Oncology Group 0915 protocol. Treatment delivery time and total monitor units were analyzed to compare the efficiency of treatment delivery. Statistical comparisons were performed using the Wilcoxon signed-rank test (*P<* 0.05).

**Results:**

For the PTV, there was no significant difference in the CI or HI between C-arm LINAC and O-ring LINAC plans. For organs-at-risk, all plans met the criteria for dose constraint. There was a significant difference between C-arm LINAC and O-ring LINAC plans except in the spinal cord. Treatment delivery time was 92% longer for C-arm LINAC plans than for O-ring LINAC plans. The total MU value for C-arm LINAC plans was 9.6% higher than that for O-ring LINAC plans.

**Conclusion:**

We verified the feasibility of fast 4DCT-based O-ring LINAC treatment for patients with average respiratory amplitude< 0.5 cm and who cannot endure long treatment times due to poor performance status in lung 4D-SBRT.

## 1 Introduction

Stereotactic body radiotherapy (SBRT) is a targeted treatment for early-stage, medically inoperable non-small-cell lung cancer (stages I and II) (1). The Radiation Therapy Oncology Group (RTOG) reported a 3-year overall survival rate of 55% and a local control rate of over 90% for patients with medically inoperable stage I disease undergoing lung SBRT (2). Unlike conventional radiation treatment, SBRT can achieve a highly biologically effective dose by delivering large doses to well-defined targets in small fractions (3). Accordingly, SBRT has been reported to exhibit excellent biological effectiveness in terms of local tumor control and acceptable levels of late complications (4). To optimize outcomes, SBRT must be precisely localized to the target, the conformation of the target must be verified, and the dose fall-off outside the target region must be determined. In addition, calculations must accurately and reproducibly account for the movement of the target due to organ movement, such as those involved in respiration or related to patient positioning (5).

Some studies reported that when the dosimetric differences between non-gating and specific phase-based gating plans were analyzed after dividing according to the criteria of specific tumor motion, the larger the tumor motion, the more dosimetric benefits for organs-at-risk (OARs) (6, 7). Heo et al. reported that gating plans were dosimetric benefits compared with non-gating plans when the average respiratory amplitude in phase 20–70% was less than 0.5 cm at the time (8). However, Fox et al. reported that the treatment delivery efficiency of specific phase-based gating plans was lower than that of non-gating plans (9).

As patients with poor performance status cannot endure long treatment times, leading to unplanned and prolonged radiation treatment or discontinuation of treatment. Acute radiation-related toxicities such as dyspnea, cough, chest pain, and pneumonitis can occur in lung four-dimensional (4D)-SBRT. Acute radiation-related toxicity can also lead to unplanned treatment breaks. Moreover, prolonged radiation treatment appears to negatively affect survival for patients. The median overall survival rates were significantly worse for patients with prolonged radiation treatment time than in those with standard radiation treatment time (18.6 vs. 22.7 months, *P*< 0.0001) (10).

In the present study, we aimed to retrospectively verify the feasibility of fast four-dimensional computed tomography (4DCT)-based O-ring linear accelerator (LINAC) treatment in patients with average respiratory amplitude< 0.5 cm and who are unable to endure long treatment times due to poor performance status by comparing dosimetric differences and the efficiency of treatment delivery between C-arm LINAC and O-ring LINAC plans in lung 4D-SBRT.

## 2 Materials and methods

### 2.1 Patient characteristics

The current study was approved by the Institutional Review Board (IRB No. ED17317) at Korea University Anam Hospital in Seoul, Korea. According to Heo et al., we selected the patients if they presented an average respiratory amplitude of 0.5 cm or less (8). A total of 38 patients with lung cancer who completed C-arm LINAC-based lung 4D-SBRT were included in this study. The average respiratory amplitude was 0.5 cm (0.03–1.87 cm) at phase values of 0–90% and 0.34 cm (0.03–0.91 cm) at phase values of 20–70% when obtained from the respiratory amplitude distribution in the contouring mode of the Eclipse TPS (version 15.6; Varian Medical Systems, Palo Alto, CA, USA). Planning target volumes (PTVs) were obtained from the dose-volume histograms (DVHs) for each patient. The average PTV was 42.62 cc (11.10–115.10 cc) at phase values of 0–90% and 37.48 cc (9.00–103.00 cc) at phase values of 20–70%.

The mean patient age was 73 years (52–86); 25 were men (65.8%) and 13 were women (34.2%) (**Table 1**). The numbers of patients with American Joint Committee on Cancer (AJCC) stages IA, IB, IIB, and lung metastasis were 16 (42.1%), 2 (5.2%), 2 (5.3%), and 18 (47.4%), respectively. Tumor locations in each patient were noted as follows: 6 (15.8%) in the left lower lobe (LLL), 14 (36.8%) in the left upper lobe (LUL), 11 (28.9%) in the right lower lobe (RLL), 1 (2.8%) in the right middle lobe (RML), and 6 (15.8%) in the right upper lobe (RUL).

**Table 1:** Patient characteristics.

		[Mean (range)]
Patient characteristics		Patients (n = 38)
Age (years)		73 (52–86)
Sex	Male	25 (65.8%)
	Female	13 (34.2%)
AJCC stage	Stage IA	16 (42.1%)
	Stage IB	2 (5.2%)
	Stage IIB	2 (5.3%)
	Lung metastasis	18 (47.4%)
Tumor location	LLL	6 (15.8%)
	LUL	14 (36.8%)
	RLL	11 (28.9%)
	RML	1 (2.8%)
	RUL	6 (15.8%)
Average respiratory amplitude (cm)	Phase 0–90%	0.51 (0.03–1.87)
	Phase 20–70%	0.34 (0.03–0.91)
PTV (cc)	Phase 0–90%	42.62 (11.10–115.10)
	Phase 20–70%	37.48 (9.00–103.00)

AJCC, American Joint Committee on Cancer; LLL, left lower lobe; LUL, left upper lobe; RLL, right lower lobe; RML, right middle lobe; RUL, right upper lobe; PTV, planning target volume.

### 2.2 4DCT acquisition and target definition

The 4DCT images were acquired using a Big Bore CT scanner (Philips Healthcare, Cleveland, OH, USA) and sorted into 10 respiratory phases labeled from 0 to 90%. For each patient, the target and organs (ipsilateral lung, contralateral lung, heart, spinal cord, and esophagus) were contoured in the 50% phase and deformably propagated across phases. Target organs and OARs were delineated in accordance with our standard protocol.

### 2.3 Planning conditions for C-arm LINAC plans

C-arm LINAC plans were based on 4DCT data obtained at phase values ranging from 20 to 70% using a C-arm LINAC (VitalBeam^®^, Varian Medical Systems, Palo Alto, CA, USA) in Eclipse TPS. C-arm LINAC plans relied on a Millennium 120 MLC with a leaf thickness of 5 mm and a 6 MV flattening-filter-free (FFF) beam. For the volumetric modulated arc therapy (VMAT) technique, the gantry was rotated at an angle of 0–180° by a 2-half arc. The prescription dose of 4,800 cGy was delivered in 4 fractions and optimized such that 95–105% of the prescription dose was delivered to the target. All plans were calculated using the advanced AurosXB system (Varian Eclipse TPS, version 15.6) with heterogeneity correction using the Photon Optimizer (PO) MLC algorithm. Dose constraints based on the RTOG 0915 protocol were applied for the ipsilateral lung, contralateral lung, heart, spinal cord, and esophagus for OARs (ipsilateral, contralateral lung: D_1000cc_< 12.4 Gy, D_1500cc_< 11.6 Gy, heart: D_max_< 34.0 Gy, V_28Gy_< 15 cc, spinal cord: D_max_< 26 Gy, V_13.6Gy_< 1.2 cc, V_20.8Gy<_ 0.35 cc, esophagus: D_max_< 30 Gy, V_18.8Gy_< 5 cc) (1).

### 2.4 Planning conditions for O-ring LINAC plans

O-ring LINAC plans were retrospectively established based on 4DCT data for phase values of 0–90% using an O-ring LINAC. O-ring LINAC plans used a dual-layer MLC with a leaf thickness of 5 mm. O-ring LINAC has two times faster MLC speed (5.0 cm/s vs. 2.5 cm/s), and four times faster gantry speed (4 RPM vs. 1 RPM) compared with C-arm LINAC (**Table 2**).

**Table 2:** Treatment delivery efficiency parameters of O-ring LINAC and C-arm LINAC.

Parameters	O-ring LINAC (11)	C-arm LINAC (12)
Gantry speed	4 RPM	1 RPM
MLC speed	5.0 cm/s	2.5 cm/s
Maximum dose rate (MU/min)	800 MU/min	1400 MU/min

### 2.5 Analysis of dosimetric differences

The dosimetric differences between C-arm LINAC and O-ring LINAC plans were analyzed using dosimetric parameters (**Table 3**). PTVs were compared using the conformity index (CI), homogeneity index (HI), and gradient measurement (GM) (13–15). Notably, because the dose gradient should be sharp in all directions around the target, the dose fall-off must be evaluated to ensure precise delivery of the high dose to the tumor and minimize damage to OARs. The GM, one of the dosimetric parameters used to evaluate dose fall-off, was calculated as the difference in radius between the 50% prescription isodose volume and the 100% prescription isodose volume, which reflects intermediate dose spillage (15). OARs were analyzed by calculating D_1000cc_ and D_1500cc_ for the ipsilateral and contralateral lungs; D_max_ and V_28Gy_ for the heart; D_max_, V_13.6Gy_, and V_20.8Gy_ for the spinal cord; and D_max_ and V_18.8Gy_ for the esophagus in accordance with RTOG 0915 methods (1).

**Table 3:** Dosimetric parameters used in this study, with corresponding formula in terms of PTV.

Dosimetric parameters for PTV	Formula (13–15)
Conformity index (CI)	$\text{CI}=\frac{\text{PTV}\times \text{PIV}}{{\text{PTV}}_{\text{PIV}}^{2}}$
Homogeneity index (HI)	$\text{HI}=\frac{{\text{D}}_{2\%}-{\text{D}}_{98\%}}{{\text{D}}_{\text{mean}}}$
Gradient measure (GM)	$\text{GM}=\sqrt[{3}]{\frac{3{\text{V}}_{50\%{\text{R}}_{\text{x}}}}{4\text{π}}}-\sqrt[{3}]{\frac{3{\text{V}}_{\text{Rx}}}{4\text{π}}}$

PTV, planning target volume; PIV, prescription isodose volume; PTV_PIV_, planning target volume receiving the prescribed dose or more; D_2%_, minimum dose to 2% volume of the PTV; D_98%_, minimum dose to 98% volume of the PTV; D_mean_, mean dose of the PTV; V_50%Rx_, volumes receiving a dose equal to or greater than the 50% prescription dose; V_Rx_, volumes receiving a dose equal to or greater than the 100% prescription dose.

The necessary parameters were extracted for dose analysis by creating a database using Python (version 3.9.6) after exporting DVH data for the 38 patients from the Eclipse TPS. The ipsilateral and contralateral lungs were defined based on the tumor location. When the tumor location was the right lung, the right lung was defined as the ipsilateral lung, while the left lung was defined as the contralateral lung. The mean ± standard deviation (SD) was used to analyze dosimetric differences between C-arm LINAC and O-ring LINAC plans.

### 2.6 Comparison of treatment delivery efficiency

**Figure 1:**
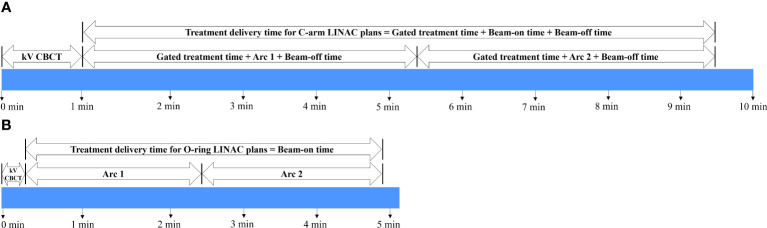
Timeline for the acquisition of treatment delivery efficiency for 38 patients undergoing lung SBRT. Workflow of integrated kV CBCT acquisition and treatment delivery time for **(A)** C-arm LINAC and **(B)** O-ring LINAC plans. Gated treatment time: beam-off time to get a respiratory signal. Beam-on time: time reaching below the threshold of the predefined phase. Beam-off time: time reaching above the threshold of the predefined phase. SBRT, stereotactic body radiotherapy; CBCT, cone beam computed tomography.

Treatment delivery time and total monitor unit (MU) values obtained from the Eclipse TPS system QA summary were analyzed to compare the efficiency of treatment delivery between C-arm LINAC and O-ring LINAC plans. The treatment delivery time of O-ring LINAC plans was recorded as beam-on time (**Figure 1A**). The treatment delivery time of the C-arm LINAC plan was recorded as the sum of the gated treatment time, beam-on time, and beam-off time (**Figure 1B**). MU was defined as the sum of MU1 of Arc 1 and MU2 of Arc 2. The kV CBCT acquisition times were 16.6 s and 60 s when O-ring LINAC and C-arm LINAC were used, respectively. The kV CBCT acquisition time was excluded from the treatment delivery time.

#### 2.6.1 Statistical analysis

Dosimetric differences and the efficiency of treatment delivery (mean ± standard deviation) for the O-ring LINAC and C-arm LINAC plans were compared using Wilcoxon signed-rank tests (*P*< 0.05). Statistical analyses were performed using Statistical Package for the Social Sciences software ver. 26.0 (IBM, Armonk, NY, USA).

## 3 Results

### 3.1 Analysis of dosimetric differences

#### 3.1.1 Comparison of isodose distribution and average DVH

**Figure 2:**
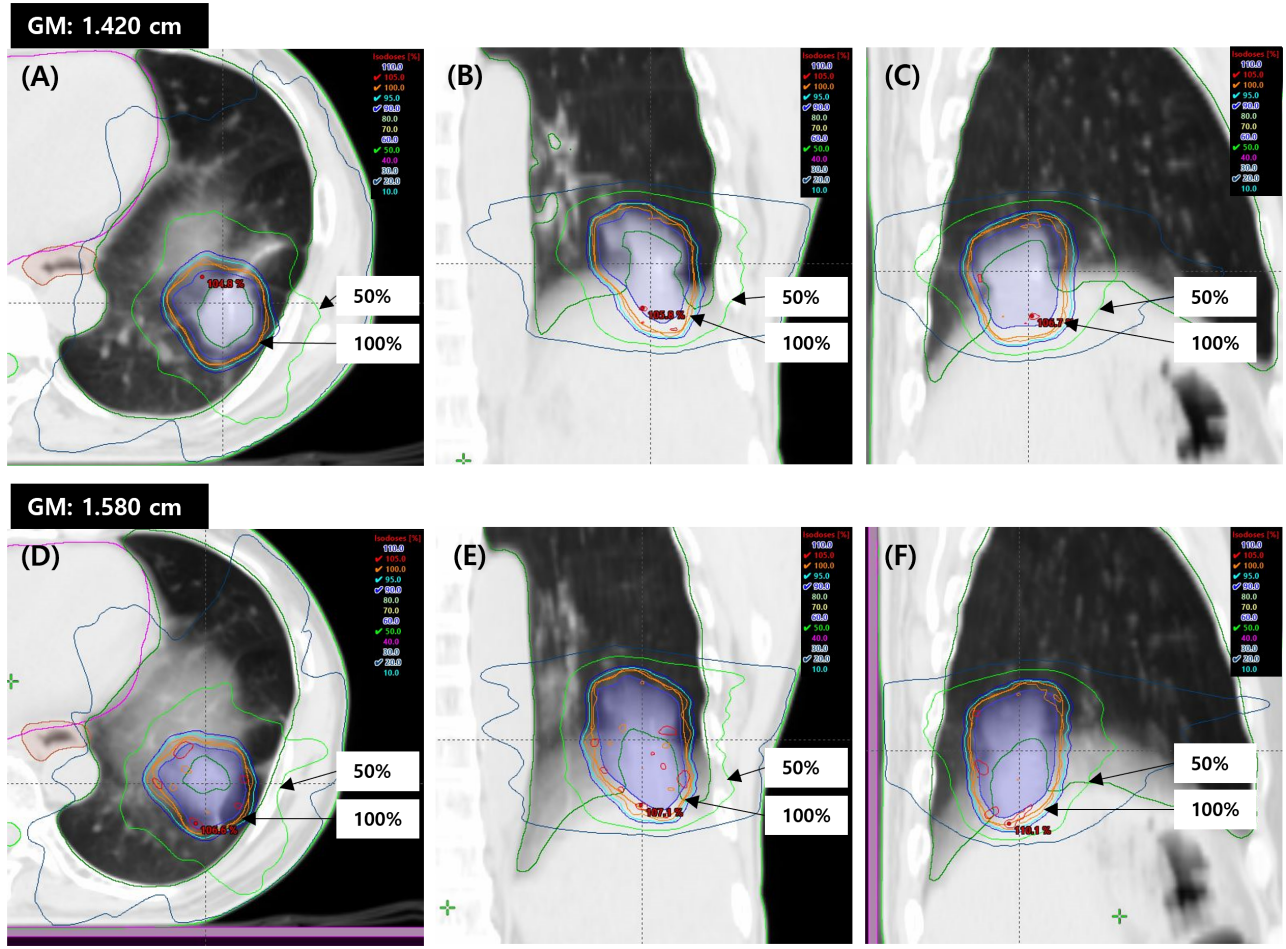
Dose distributions for a representative case (Patient 1) in the comparison between C-arm LINAC and O-ring LINAC plans for lung SBRT. Representative isodose distributions of the C-arm LINAC plan in the **(A)** axial, **(B)** coronal, and **(C)** sagittal planes. Representative isodose distributions of the O-ring LINAC plan in the **(D)** axial, **(E)** coronal **(F)** sagittal planes. Representative GM values are shown for the O-ring LINAC and C-arm LINAC plans. The isodose lines represent 105% (red), 100% (orange), 95% (cyan), 90% (blue), 50% (green), and 20% (dark blue). SBRT, stereotactic body radiotherapy; GM, gradient measurement.

**Figure 3:**
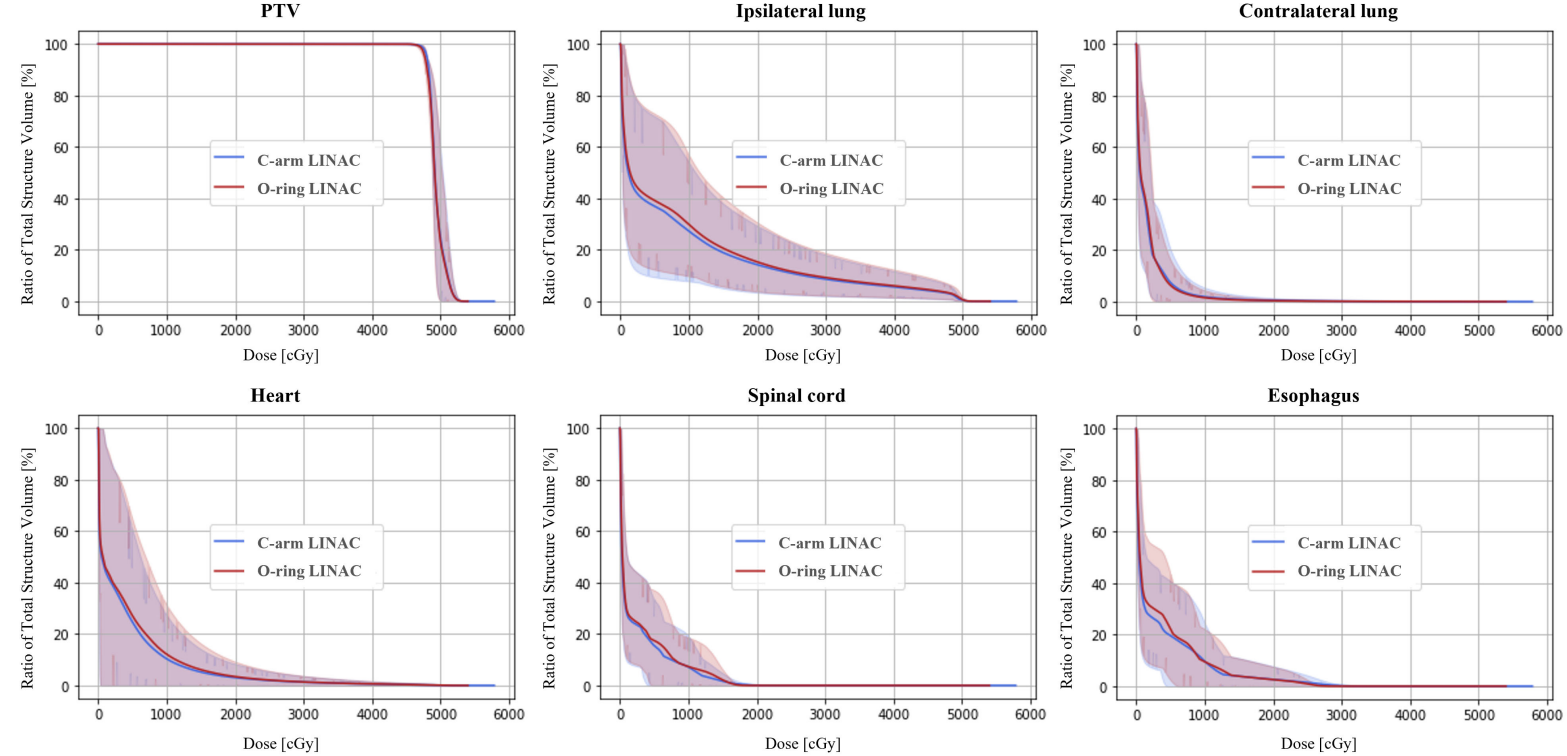
Average dose-volume histogram (DVH) for the planning target volume (PTV) and organs-at-risk (OARs). The solid line and shaded bands represent the average and minimum-maximum range of the DVHs across all 38 patients who received SBRT.

The isodose distributions in the axial, coronal, and sagittal planes were compared between the C-arm LINAC and O-ring LINAC plans (**Figure 2**). When the coronal and sagittal planes of the C-arm LINAC plans were compared with those of the O-ring LINAC plans, the 50% isodose line became smaller as the PTV volume decreased. The average DVH for target coverage and doses delivered to OARs were also compared between O-ring LINAC and C-arm LINAC plans (**Figure 3**). Both plans resulted in similar target coverage and met RTOG 0915-based dose constraints for OARs.

#### 3.1.2 Comparison of dosimetric parameters

In the analysis of PTV and OARs volumes, significant differences were noted between the C-arm LINAC and O-ring LINAC plans (all *P*< 0.05) (**Table 4**). The PTV for C-arm LINAC plans was 12.1% smaller than that for O-ring LINAC plans (37.484 ± 22.544 cc, 42.621 ± 24.867 cc, *P*< 0.001). The ipsilateral and contralateral lung volumes were 6.0% and 5.6% lower for C-arm LINAC plans than for O-ring LINAC plans, respectively (*P*< 0.001). The heart, esophagus, and spinal cord volumes were 4.6%, 8.9%, and 2.6% lower for C-arm LINAC plans than for O-ring LINAC plans, respectively.

**Table 4:** Dosimetric differences between C-arm LINAC and O-ring LINAC plans.

				[Mean ± SD]
Structure	Dosimetric parameter	C-arm LINAC	O-ring LINAC	*P*
PTV	**Volume (cc)**	**37.484 ± 22.544**	**42.621 ± 24.867**	< 0.001^*^
	CI	1.335 ± 0.174	1.337 ± 0.137	0.199
	HI	0.939 ± 0.016	0.939 ± 0.015	0.538
	**GM (cm)**	**1.381 ± 0.222**	**1.528 ± 0.206**	< 0.001^*^
Ipsilateral lung	**Volume (cc)**	**1,551.326 ± 482.451**	**1,650.466 ± 497.858**	< 0.001^*^
	**D_1000cc_ (cGy)**	**34.755 ± 102.480**	**50.780 ± 132.061**	< 0.001^*^
	**D_1500cc_ (cGy)**	**2.666 ± 7.926**	**6.641 ± 14.598**	< 0.001^*^
Contralateral lung	**Volume (cc)**	**1,546.229 ± 507.308**	**1,638.082 ± 532.270**	< 0.001^*^
	**D_1000cc_ (cGy)**	**20.671 ± 37.433**	**30.902 ± 50.234**	< 0.001^*^
	**D_1500cc_ (cGy)**	**6.979 ± 13.507**	**10.299 ± 18.258**	< 0.001^*^
Heart	**Volume (cc)**	**893.050 ± 181.171**	**938.637 ± 181.232**	< 0.001^*^
	**D_max_ (cGy)**	**39.945 ± 34.947**	**43.042 ± 35.736**	**0.020**
	**V_20Gy_ (cc)**	**2.809 ± 8.099**	**3.926 ± 9.589**	**0.008**
Spinal cord	**Volume (cc)**	**42.724 ± 10.391**	**43.866 ± 10.339**	< 0.001^*^
	D_max_ (cGy)	22.713 ± 7.671	23.292 ± 7.667	0.429
	V_13.6Gy_ (cc)	0.180 ± 0.618	0.262 ± 0.928	0.594
	V_20.8Gy_ (cc)	0.001 ± 0.008	0.000 ± 0.000	0.317
Esophagus	**Volume (cc)**	**51.642 ± 18.273**	**56.697 ± 18.974**	< 0.001^*^
	**D_max_ (cGy)**	**26.792 ± 11.210**	**28.792 ± 9.348**	**0.005**
	**V_18.8Gy_ (cc)**	**0.421 ± 0.972**	**0.637 ± 1.295**	**0.001**

^*^P< 0.001: since P< 0.001, there is statistically strong evidence to reject H_0_ and concluded that dosimetric parameters differ between C-arm LINAC and O-ring LINAC plans, PTV: planning target volume; CI: conformity index, HI: homogeneity index, GM: gradient measurement, D_1000cc_: dose received by 1,000 cc volume of a structure, D_1500cc_: dose received by 1,500 cc volume of a structure, V_28Gy_: absolute volume receiving 28 Gy, D_max_: maximum dose of a structure, V_13.6Gy_: absolute volume receiving 13.6 Gy, V_20.8Gy_: absolute volume receiving 20.8 Gy, V_18.8Gy_: absolute volume receiving 18.8 Gy. Values with statistically significant differences (p-value<0.05) between O-ring LINAC and C-arm LINAC plans are shown in bold.

In the analysis of PTVs, there were no statistically significant differences between the CI and HI (all *P* > 0.05). However, the difference in radius between the 50% isodose and 100% isodose lines for C-arm LINAC plans was reduced when compared with that for O-ring LINAC plans. In addition, the GM was 9.6% lower for C-arm LINAC plans than for O-ring LINAC plans (1.381 ± 0.222 cm and 1.528 ± 0.206 cm, *P*< 0.001) (**Table 4**, **Figure 3**). For OARs, all plans met the criteria for dose constraints based on the RTOG 0915 protocol (**Table 4**). Within the dose constraint criteria, there were no significant differences except in the spinal cord. The D_1000cc_ and D_1500cc_ values for the ipsilateral lung for C-arm LINAC plans were reduced by 31.6% and 59.9% when compared with those for O-ring LINAC plans, respectively (D_1000cc_: 34.755 ± 102.480 cGy, 50.780 ± 132.061 cGy, *P*< 0.001, D_1500cc_: 2.666 ± 7.926 cGy, 6.641 ± 14.598 cGy, *P*< 0.001). The D_1000cc_ and D_1500cc_ values for the contralateral lung for C-arm LINAC plans were reduced by 33.1% and 32.2%, respectively, when compared with those for O-ring LINAC plans (D_1000cc_: 20.671 ± 37.433 cGy, 30.902 ± 50.234 cGy, *P*< 0.001, D_1500cc_: 6.979 ± 13.507 cGy, 10.299 ± 18.258 cGy, *P*< 0.001). The D_max_ and V_20Gy_ values for the heart for C-arm LINAC plans were 7.1% and 28.5% lower than those for O-ring LINAC plans, respectively. The D_max_ and V_20Gy_ values for the esophagus for C-arm LINAC plans were reduced by 6.9% and 33.9%, respectively, when compared with those for O-ring LINAC plans. The D_max_, V_13.6Gy_, and V_20.8Gy_ values for the spinal cord for C-arm LINAC plans were reduced by 2.5%, 31.3%, and 0.0%, respectively, when compared with those for the O-ring LINAC plans; however, these differences were not significant.

### 3.2 Comparison of treatment delivery efficiency

**Figure 4:**
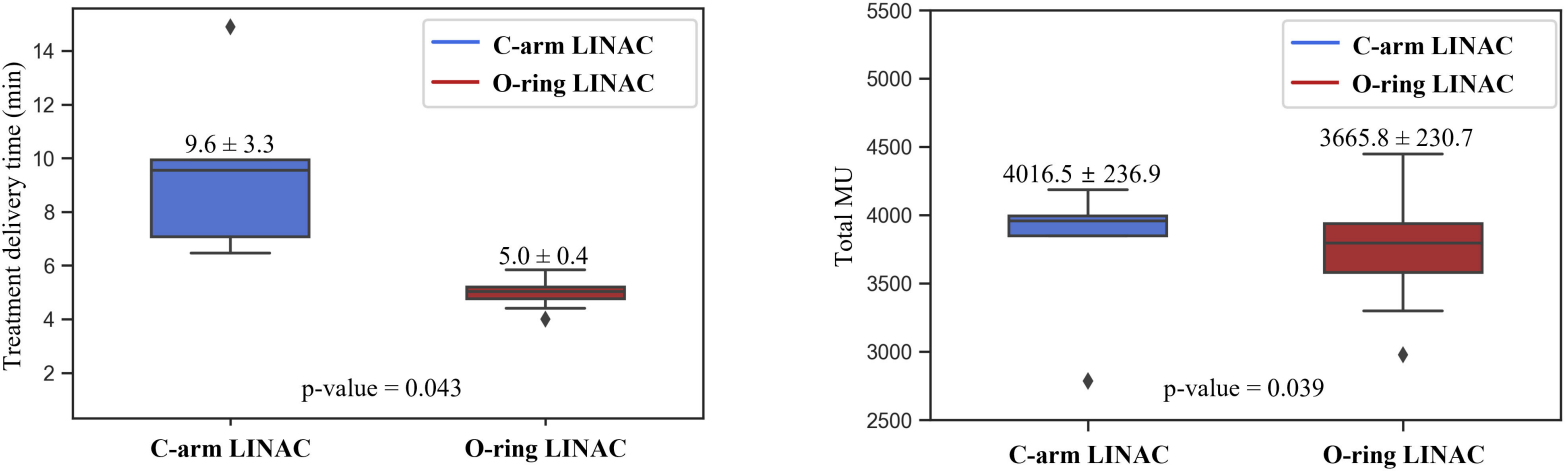
Comparison of treatment delivery time between C-arm LINAC and O-ring LINAC plans.

For treatment delivery efficiency, there were significant differences between the C-arm LINAC and O-ring LINAC plans (all *P*< 0.05) (**Figure 4**). Treatment delivery time was 92% longer for C-arm LINAC than for O-ring LINAC plans (9.6 ± 3.3 min, 5.0 ± 0.4 min, *P* = 0.043). The total MU value was 9.6% higher for C-arm LINAC plans than for O-ring LINAC plans (4016.5 ± 236.9, 3665.8 ± 230.7, *P* = 0.039).

## 4 Discussion

In this study, we tried to retrospectively verify that the feasibility of fast 4DCT-based O-ring LINAC treatment for patients with an average respiratory amplitude< 0.5 cm and who cannot endure long treatment times due to poor performance status in lung 4D-SBRT.

In our study, patients enrolled in this study were selected based on the average respiratory amplitude is< 0.5 cm in the full phase by Heo et al. They analyzed dosimetric differences between plan_0-90%_, plan_20-70%,_ and plan_40-60%_ in 40 patients treated with lung 4D-SBRT. Compared with plan_0-90%_, D_1000cc_ of plan_20-70%,_ and plan_40-60%_ were reduced for ipsilateral lung (1.36 ± 1.58 Gy, 0.98 ± 0.96 Gy, 0.77 ± 0.79 Gy, *P*-value< 0.05). However, all plans met the criteria for dose constraints based on the RTOG protocol. The average respiratory amplitude in phase 20–70% was< 0.5 cm at the time (8). In addition, in our study, we included patients treated C-arm LINAC plans using C-arm LINAC, and we retrospectively established O-ring LINAC plans using a large-scale database (38 patients). A larger sample size is needed to verify statistically significant differences. Pokhrel et al. reported when compared dosimetric differences between non-gating plans in patients treated with lung SBRT, clinical follow-up results are considered essential (16).

Some studies compared dosimetric differences between specific phase-based gating and free-breathing-based non-gating plans that divided two groups as 0.5 cm< tumor motion< 1 cm and tumor motion > 1 cm. Prunaretty et al. reported that when the motion was > 1 cm, the HI of PTV was 6.67% higher for gating plans than for free-breathing plans. When the tumor motion was< 1 cm, the HI of PTV was 16.67% higher for gating plans than for free-breathing plans. In addition, when the tumor motion was > 1 cm, the V_20Gy_ values of ipsilateral lung were 49.29% lower for gating plans than for free-breathing plans. When the tumor motion was< 1 cm, the V_20Gy_ of ipsilateral lung were 21.18% lower than for free-breathing plans (7). All plans met the ROSEL constraints (17). Jang et al. reported that when the tumor motion was > 1 cm, the V_20Gy_ values of the ipsilateral lung were 0.41% and 1.11% lower for gating_GW50%_ (5 phases) and gating_GW25%_ (3 phases) plans, respectively, when compared with non-gated plans. When the tumor motion was< 1 cm, the V_20Gy_ values of the ipsilateral lung were 0.31% and 0.42% lower for gating_GW50%_ and gating_GW25%_ plans, respectively, when compared with non-gated plans. However, in our study, patients with average respiratory amplitude< 0.5 cm were selected. The average respiratory amplitude was 0.51 cm (0.03–1.87 cm) at phase values of 0–90% and 0.34 cm (0.03–0.91 cm) at phase values of 20–70%, respectively. For PTV, the CI and HI were 0.15% and 0% higher for C-arm LINAC plans than for O-ring LINAC plans, respectively. As in previous studies, there were no statistically significant differences between the CI and HI (all *P* > 0.05). For OARs, the D_1000cc_ and D_1500cc_ values of the ipsilateral lung were also 31.6% and 59.9% lower for C-arm LINAC plans than for O-ring LINAC plans, respectively. In addition, there were significant differences between the plans (all *P*< 0.001). However, all plans met the criteria for dose constraints based on the RTOG 0915 protocol.

Fox et al. analyzed the factors affecting the treatment delivery time during gating plans established based on specific phase in 15 patients treated with gating plans and 13 patients treated with non-gated plans. Thy reported that the beam-on time of gating plans increased by 5.5 times (range 1.2–12.2) than that of the non-gated treatment (9). Our study found similar results. Treatment delivery time was 92% longer for C-arm LINAC than for O-ring LINAC plans. We verified the feasibility of fast 4DCT-based O-ring LINAC treatment for patients with an average respiratory amplitude< 0.5 cm and who cannot endure long treatment times due to poor performance status in lung 4D-SBRT.

Wang et al. reported that tumor motion variation was higher in the upper lobe compared with the lower lobe (10 ± 4.8 mm vs. 2.3 ± 2.2 mm) (18). Therefore, considering the tumor location, the feasibility of a fast respiratory motion management decision support guideline for lung SBRT in patients with poor performance status must be considered in future studies.

## 5 Conclusion

We verified the feasibility of fast 4DCT-based O-ring LINAC treatment for patients with an average respiratory amplitude< 0.5 cm and who cannot endure long treatment times due to poor performance status in lung 4D-SBRT. Compared with OARs, both plans were acceptable according to the RTOG-0915 protocol, but no significant differences between O-ring LINAC and C-arm LINAC plans. However, treatment delivery time was 92% longer for C-arm LINAC plans than for O-ring LINAC plans.

## Data availability statement

The original contributions presented in the study are included in the article/supplementary material. Further inquiries can be directed to the corresponding authors.

## Ethics statement

The studies involving humans were approved by Korea University Anam Hospital Institutional Review Board. The studies were conducted in accordance with the local legislation and institutional requirements. The participants provided their written informed consent to participate in this study.

## Author contributions

EH: Conceptualization, Data curation, Formal Analysis, Investigation, Methodology, Software, Validation, Visualization, Writing – original draft. MK: Formal Analysis, Software, Validation, Visualization, Writing – review & editing. CP: Formal Analysis, Software, Validation, Visualization, Writing – review & editing. KC: Writing – review & editing. KK: Writing – review & editing. JS: Writing – review & editing. YP: Writing – review & editing. CK: Writing – review & editing. NL: Writing – review & editing. SL: Conceptualization, Investigation, Supervision, Writing – review & editing, Methodology.
